# Institution specific risk factors for 30 day readmission at a community hospital: a retrospective observational study

**DOI:** 10.1186/1472-6963-14-40

**Published:** 2014-01-27

**Authors:** Lee Park, Danielle Andrade, Andrew Mastey, James Sun, LeRoi Hicks

**Affiliations:** 1 Hospital Medicine Unit, Division of General Internal Medicine, Massachusetts General Hospital, 50 Staniford St, Suite 503B, Boston, MA 02114, USA; 2 Decision Support Group, Newton-Wellesley Hospital, 2014 Washington St, Newton, MA 02462, USA; 3 Comparative Data and Informatics Group, University Health System Consortium, 155 North Wacker Drive Chicago, Chicago, IL 60606, USA; 4 Harvard College, Harvard University 386 Leverett Mail Center, Cambridge, MA 02138, USA; 5 Division of Hospital Medicine, University of Massachusetts Medical Center, 119 Belmont St, Worcester, MA 01605, USA; 6 50 Staniford St., Suite 503B, Boston, MA 02115, USA

**Keywords:** Readmission, Hospital quality, Risk factors

## Abstract

**Background:**

As of October 1, 2012, hospitals in the United States with excess readmissions based on the Centers for Medicare and Medicaid Services (CMS) risk-adjusted ratio began being penalized. Given the impact of high readmission rates to hospitals nationally, it is important for individual hospitals to identify which patients may be at highest risk of readmission. The objective of this study was to assess the association of institution specific factors with 30-day readmission.

**Methods:**

The study is a retrospective observational study using administrative data from January 1, 2009 through December 31, 2010 conducted at a 257 bed community hospital in Massachusetts. The patients included inpatient medical discharges from the hospitalist service with the primary diagnoses of congestive heart failure, pneumonia or chronic obstructive pulmonary disease. The outcome was 30-day readmission rates. After adjusting for known factors that impact readmission, provider associated factors (i.e. hours worked and census on the day of discharge) and hospital associated factors (i.e. floor of discharge, season) were compared.

**Results:**

Over the study time period, there were 3774 discharges by hospitalists, with 637 30-day readmissions (17% readmission rate). By condition, readmission rates were 19.6% (448/2284) for congestive heart failure, 13.0% (141/1083) for pneumonia, and 14.7% (200/1358) for chronic obstructive lung disease. After adjusting for known risk factors (gender, age, length of stay, Elixhauser sum score, admission in the previous year, insurance, disposition, primary diagnosis), we found that patients discharged in the winter remained significantly more likely to be readmitted compared to the summer (OR 1.54, p = 0.0008). Patients discharged from the cardiac floor had a trend toward decreased readmission compared a medical/oncology floor (OR 0.85, p = 0.08). Hospitalist work flow factors (census and hours on the day of discharge) were not associated with readmission.

**Conclusions:**

We found that 30 day hospital readmissions may be associated with institution specific risk factors, even after adjustment for patient factors. These institution specific risk factors may be targets for interventions to prevent readmissions.

## Background

In the United States, nearly one-fifth of all Medicare beneficiaries are readmitted within 30 days after discharge, at an annual cost of $17.4 billion [1]. As of October 1, 2012, hospitals with excess readmissions based on the Centers for Medicare and Medicaid Services (CMS) risk-adjusted ratio began being penalized up to 1% of reimbursement for inpatient services [2-4] and by the fiscal year 2015, the penalty cap will increase to 3% [5,6] Furthermore, Medicare’s Hospital Compare website [7] will soon expand its current publication of readmission rates for selected diagnoses to include all patient readmissions [5].

Given the impact of high readmission rates to hospitals, it is important for individual hospitals to identify which patients may be at highest risk of being readmitted. Certain risk factors, such as patient age, race, diagnoses, length of stay (LOS), comorbidities, insurance, disposition, and prior hospitalizations, are well-documented [8-14]. However, while there is significant pressure to decrease readmission rates, studies have found that only a certain percentage of readmissions are preventable [15], models developed to predict readmissions have generally been poor [16,17], and interventions on a broad scale have largely been unsuccessful [18-20].

Each hospital has its own structure and processes, and while patient factors certainly impact the risk of readmission, we hypothesized that predictions models and interventions may be challenging to apply broadly because of the heterogeneity of hospitals. In this study, we examined various risk factors for readmission for patients with the diagnoses of congestive heart failure (CHF), pneumonia (PNA), and chronic obstructive pulmonary disease (COPD) in a community hospital setting in the United States. We hypothesized that patient related risk factors for readmission would be comparable to previous studies. In addition, we hypothesized that there may be factors related to the structure of the hospitalist service and/or hospital that may affect readmission.

## Methods

### Setting

Newton-Wellesley Hospital (NWH) is a 257 bed community hospital (a hospital that is not an academic medical center though may be affiliated with one) with a mix of housestaff (resident or trainee) and non-housestaff covered medical services. For the two years from January 1, 2009 to December 31, 2010, there were 10,816 inpatient medical discharges with most patients being cared for by hospitalists. Medical patients are admitted to one of six teams - four housestaff and two non-housestaff teams. The housestaff teams are staffed by residents with a hospitalist attending. The non-housestaff teams have a hospitalist attending paired with a physician assistant. The medical service is regionalized to three floors: general medicine, general medicine/oncology and cardiac/telemetry floor. Medical patients are assigned to one of these three floors based on diagnosis and bed-availability, though occasionally medical patients are admitted to non-medical floors. Hospitalists are on-service for one week periods, with new attendings switching onto service each Wednesday. Housestaff are on-service for two to four week periods, with new housestaff switching onto service on specific Thursdays. The study design and procedures were approved by the NWH Institutional Review Board.

### Study design

We collected data regarding potential patient, provider and hospital associated predictors of readmission through a survey of hospitalists and a preliminary manual chart review of readmitted and non-readmitted patients. We then performed a larger retrospective observational study using administrative data.

### Survey of hospitalists

From May 2011 to June 2011, we surveyed hospitalists at NWH through an email questionnaire. We first emailed providers with a survey questionnaire for two to three patients whom they had discharged from January 1, 2011 to April 31, 2011 with the primary diagnosis of CHF or PNA and readmitted within 30 days. We requested that the providers fill out the survey and return to us via email. We sent another request 2 weeks after the initial survey request. Of 20 hospitalists, 13 responded (response rate of 65%) regarding 21/34 (62%) patients. The goal of our survey was to ascertain provider reporting of patient, provider, and hospital level characteristics that may have led to readmission. Hospitalists provided insight that the complexity of the patient’s medical illnesses, number and complexity of the discharge medication list, hospitalist census and hours worked on the day of discharge, day of the week of discharge, and the hospital census on the day of discharge may contribute to readmissions.

### Preliminary data analysis

The patients selected for the survey were included in a preliminary analysis from a chart review of 101 patients (the 34 readmitted patients above as well as 67 non-readmitted patients) discharged between January 1, 2011 to April 31, 2011 with CHF or PNA. Based on answers from the survey above, we opted to include data regarding hospitalist census and hours worked on the day of discharge, the day of the week of discharge, focusing on switch days (the first day on service for either housestaff or hospitalists), and information on hospital census on the day of discharge into the larger analysis. We were unable to obtain information on the number or type of discharge medications from the administrative databases and this information was ultimately not included in the larger analysis. The preliminary analysis was done to verify that the electronic data accurately reflected data in the chart review and to confirm which variables could be accurately gleaned from the electronic administrative data.

### Retrospective observational study

We examined data from inpatient medical discharges with a primary diagnosis of CHF, PNA or COPD from January 1, 2009 to December 31, 2010 at NWH. We collected data regarding patient and hospital factors from the hospital’s administrative databases. We collected data on provider factors from the hospitalist group administrative records. Because the data was taken from available administrative databases, there was minimal missing data (less than 10 data points).

Patient readmissions were defined as inpatient re-hospitalization within 30 days of discharge (30-day readmission) for any reason to any service. Discharges that led to a 30-day readmission and those that did not lead to readmission were compared. We included only patient admissions that were discharged by the hospitalist service with the primary diagnoses of CHF, PNA or COPD. We chose to focus on the three diagnoses of CHF, PNA and COPD as these have been shown to be the three most common medical discharge diagnoses associated with readmissions [1] and to decrease confounding in our sample. We excluded patient admissions that were transferred to another acute care hospital, were an elective readmission for chemotherapy, or admissions where the patient expired during the index admission.

#### Variables were summarized as follows

Patient associated factors: Gender was male or female. Age and LOS were left as continuous variables. Patient morbidities based on secondary diagnosis were put into Elixhauser categories [21] and the Elixhauser categories were summed into a numerical score which reflected patient morbidity (including the principle diagnosis). The Elixhauser sum score was a continuous variable. Previous admission was a binary variable with “yes” being any admission in the previous calendar year. Disposition had two categories: home or home with services vs. rehabilitation facility, skilled nursing facility, or long term acute care facility. Insurance had three categories: Medicare, Medicaid/uninsured (grouped because of the low numbers of both Medicaid and uninsured patients in our patient population), and private insurance.

Provider associated factors: Hospitalist census and hours worked on the discharge day were left as continuous variables.

Hospital associated factors: Discharge on a switch day was a binary variable with “yes” being discharge on Wednesdays for the hospitalist switch days and specific Thursdays that reflected housestaff switch days. Service had two categories: housestaff service or non-housestaff. Month of discharge was simplified to season with four seasons: winter (December, January, February), spring (March, April, May), summer (June, July, August), and fall (September, October, November). Floor had three categories: medical/oncology, cardiac, other (non-medical). Floor census was divided into three categories: 0-20, 20-30 and >30. Medicine/surgery census (the census on the medical and surgical services) and total hospital census were left as continuous variables.

### Analysis

We compared 30-day hospital readmission with each candidate predictor in bi-variable analyses. We compared the unadjusted associations with readmission continuous variables using Students t-test with the exception of LOS where we used a Wilcoxon test. For examinations of association between hospital readmission and categorical variables, we utilized chi-square tests.

In adjusted analyses, we utilized a multivariable logistic regression model to assess which, if any, of our provider or hospital specific variables were independently associated with 30-day readmission by adjusting for variables known to impact readmission. Variables that were included into the base model included: gender, age, LOS, Elixhauser sum score, insurer, hospitalizations in the previous year, disposition and primary diagnoses (CHF, PNA or COPD). Other candidate variables were then each put into the base model individually to determine which, if any, were associated with readmissions. In total, 100% of our initial sample was included in the final multivariable model. All analyses were carried out using SAS version 9.3.

## Results

### Patient characteristics

During the two year study period, there were 4012 medical discharges with a diagnosis of CHF, PNA and/or COPD. Of the 4012 total discharges, 3774 (94%) were hospitalist discharges. Among the 3774 hospitalist discharged patients, 40.9% were men and the mean age was 76.4 years (±15.5 years SD). The mean LOS was 4.1 days (±3.4 days SD) and Elixhauser sum score was 3.9 (±1.6 SD). Regarding patient demographics, 71.3% had Medicare as their primary insurer, 15.5% had been readmitted in the previous calendar year, and 61.5% of patients were discharged home. Among our cohort, 2284 of patients (60.5%) had CHF, 1083 (28.7%) had PNA and 1358 (36.0%) had COPD.

### Hospital readmissions

Among the 3774 hospitalist discharges, 637 (17%) were readmitted within 30 days. By condition, readmission rates were 19.6% (448/2284) for CHF, 13.0% (141/1083) for PNA, and 14.7% (200/1358) for COPD.

Baseline characteristics for readmitted patients compared to non-readmitted are compared in Table 1. In unadjusted comparisons, readmitted patients were significantly older, had a longer LOS, higher Elixhauser sum score, were more likely to have been admitted in the previous calendar year and less likely to be privately insured. We also found that readmitted patients were 4% more likely to have been discharged to a facility than to home, 3.7% more likely to be discharged from the housestaff service compared to patients from the non-housestaff service, and 5% more likely to be readmitted during the wintertime compared to all other seasons (all p < 0.05) (Table 1).

**Table 1:** Comparison of patients readmitted or not within 30 days of discharge

**Characteristic**	**Readmit (N = 637)**	**Non-readmit (N = 3137)**	p-value*
**Male (N-%)**	269 (42.2)	1275 (40.6)	0.5
**Age, yrs. (mean ± SD)**	78.0 (13.7)	76.0 (15.8)	0.004
**LOS, days (mean ± SD)**	4.8 (4.3)	3.9 (3.1)	<0.0001
**Elixhauser sum score (mean ± SD)**	4.3 (1.5)	3.9 (1.7)	<0.0001
**Admission in the previous calendar year (N-%)**	139 (21.8)	445 (14.2)	<0.0001
**Disposition, home (N-%) vs. facility**	353 (15.2)	1967 (84.8)	0.0006
**Insurance**			0.001
**Medicare (N-%)**	491 (18.3)	2198 (81.7)	
**Medicaid (N-%)**	19 (17.1)	92 (82.9)	
**Private (N-%)**	127 (13.0)	847 (87.0)	
**MD census (mean ± SD)**	12.7 (2.7)	12.5 (1.8)	0.2
**MD hours (mean ± SD)**	10.7 (1.8)	10.7 (1.8)	0.9
**Discharge on switch day (N-%)**	113 (15.6)	609 (84.3)	0.3
**Service, housestaff vs. non-housestaff (N-%)**	532 (17.6)	2488 (82.4)	0.02
**Season**			0.002
**Winter (N-%)**	199 (20.6)	765 (79.4)	
**Spring (N-%)**	165 (16.6)	831 (83.4)	
**Summer (N-%)**	123 (14.4)	733 (85.6)	
**Fall (N-%)**	150 (15.7)	808 (84.3)	
**Floor**			0.7
**Med/onc (N-%)**	294 (16.9)	1449 (83.1)	
**Cardiac (N-%)**	332 (17.0)	1617 (83.0)	
**Non-medical (N-%)**	11 (13.4)	71 (86.6)	
**Floor census**			0.67
**<20 (N-%)**	131 (17.2)	632 (82.8)	
**21-30 (N-%)**	366 (16.4)	1864 (83.6)	
**>30 (N-%)**	140 (17.9)	641 (82.1)	
**Medicine/surgical census (mean ± SD)**	89 (12.5)	89 (12.8)	0.81
**Hospital census (mean ± SD)**	204 (21.7)	205 (22.8)	0.26

*P value obtained by chi-square, Student’s T-test or Wilcoxon rank sum testing.

In adjusted analysis, we found that patients discharged from the cardiac floor showed a trend toward decreased readmission (p-value > 0.05 but < 0.10) and that season of discharge was associated with readmission (Table 2).

**Table 2:** Independent odds of hospital readmission adjusting for known factors (gender, age, LOS, Elixhauser sum score, admission in the previous year, insurance, disposition, primary diagnosis)

**Covariate**	**OR (95% CI)**
MD census	1.01 (0.98, 1.04)
MD hours	0.99 (0.94, 1.04)
Discharge on a switch day	0.90 (0.72, 1.21)
Service, housestaff vs. non-housestaaff	1.10 (0.87, 1.39)
Season (referent = summer)	
Winter	1.54 (1.20, 1.98)
Spring	1.12 (0.86, 1.45
Fall	1.10 (0.84, 1.42)
Floor (referent = medical/oncology)	
Cardiac	0.85 (0.71, 1.02)
Non-medical	0.76 (0.39, 1.48)
Floor census (reference <20)	
20-30	0.84 (0.67, 1.06)
>30	0.94 (0.72, 1.23)
Medicine/surgery census	1.00 (0.99, 1.00)
Hospital census	1.00 (0.99, 1.00)

## Discussion

While patient factors certainly impact a patient’s risk of readmission, the risk may also, in part, be related to institution specific factors that impact the care of the patient during the admission. While floor was not found to be significant, it is interesting that patients who were discharged from the cardiac floor had a trend towards decreased readmission. Patients admitted to the cardiac floor at our institution, in general, have higher acuity of cardiac illnesses compared to those admitted to the medical floor. We would have expected that patients discharged from the cardiac floor would have a higher rate of readmission, rather than a trend toward decreased readmission. This tendency may be related to a variety of factors. The nurses on a cardiac floor are more familiar with CHF patients. It is easier to get daily patient weights and ins and outs on a floor that is focused on the care of cardiac patients. Patients are less likely to be discharged without seeing someone from the outpatient heart failure team. It may be that patients discharged from a cardiac floor are less likely to be readmitted because of the care they received on a floor dedicated to the care of cardiac patients.

It was unclear to us why the wintertime was associated with higher readmission rates compared to the rest of the year. There is fluctuation in the census of both the medical services and the hospital and rather than season, we would have expected hospital volume would be associated with readmission. However, neither hospitalist census, floor census nor hospital census on the day of discharge had any relation to readmission risk. All administrative census data for the hospital includes both medicine and surgery, leaving us unable to fully test the hypothesis that it is the increased medicine census that may be related to the increased risk of readmission in the wintertime.

In the adjusted analysis, we did not find that patients discharged from the housestaff service were more likely to be readmitted once we controlled for patient factors. This finding was not surprising as in our institution, housestaff patients are triaged such that they tend to have increased severity of illness compared to non-housestaff patients.

Though the inclusion of hospitalist data, we were able to study provider work flow factors and had thought that work flow factors such as the hospitalist census and work hours on the day of discharge or the day that hospitalist/housestaff switched onto service would be associated with an increased risk for readmission. However, even after adjusting for patient characteristics, we did not find that association in this study. It may be that these factors are specific to individual hospitalists and studying work flow factors as a group mitigated the individual differences.

The study does have limitations. We faced limitations with the administrative data available. For example, while we initially had hoped to include information on the number or type of discharge medications, that information was not easily available in the administrative data. We also note that this study is a single center study. While this limitation decreases the generalizability of our study to other settings, our hypothesis that there may be institution specific risk factors for readmission may be best studied in a single site setting. We were able to study hospitalist work flow factors that have not yet been studied else ware in the literature because of the single site nature of the study. Other limitations we note include that we did not include all diagnoses in our analysis, though the diagnoses we included are the most common diagnoses that are associated with readmissions [1]. We were not able to distinguish between preventable and non-preventable readmissions. We were not able to capture readmissions to other hospitals, and based on previous studies, that would account for close to 25% of readmissions [1], however, the readmission rate found in our study is close to the rate found in other studies [10,22]. We were unable to take into account patients who may have expired in the outpatient setting though we excluded patients who expired in the hospital.

## Conclusions

Given that readmissions are common among medical patients with almost a fifth of Medicare patients returning within 30 days of discharge and that CMS has started to penalize for higher risk adjusted readmission rates, it is critically important for hospitals to develop efforts focused on institutional related mediators of early readmissions. We found that factors related to the structure of the hospital (floor) and season may further contribute to readmission risk beyond specific patient-related factors found in existing literature. We expected to find provider work flow factors that affected readmission, but in our study, these factors did not appear to be related to readmission. Future studies could continue to identify institution specific risk factors and design interventions aimed at those risk factors to evaluate how those interventions impact readmissions. Institution specific risk factors for readmissions may provide a better idea of where to focus efforts to prevent readmissions and these efforts can be aimed at providers or hospital processes rather than patients. These interventions may be easier to implement and more cost effective than those aimed at entire patient populations and they may also improve the quality of care during the hospitalization.

## Abbreviations

CMS: Centers for Medicare and Medicaid services; LOS: Length of stay; CHF: Congestive heart failure; PNA: Pneumonia; COPD: Chronic obstructive pulmonary disease; NWH: Newton-Wellesley hospital.

## Competing interests

The authors declare that they have no competing interests.

## Authors’ contributions

LP conceived of the study, participated in its design and coordination including gathering the administrative and hospitalist data, data analysis and interpretation and drafted the manuscript. AD and AM participated in the designing the survey portion of the study, ensuring the integrity of the administrative data, gathering and providing the administrative and hospitalist data and drafting the manuscript. JS participated in gathering data for the survey portion of the study and drafting the manuscript. LSH conceived of the study, participated in the design and coordination and helped to draft the manuscript. All authors read and approved the final manuscript.

## Pre-publication history

The pre-publication history for this paper can be accessed here:

http://www.biomedcentral.com/1472-6963/14/40/prepub
